# The Impact of Tree Diversity on Different Aspects of Insect Herbivory along a Global Temperature Gradient - A Meta-Analysis

**DOI:** 10.1371/journal.pone.0165815

**Published:** 2016-11-11

**Authors:** Stephan Kambach, Ingolf Kühn, Bastien Castagneyrol, Helge Bruelheide

**Affiliations:** 1 Department of Community Ecology, Helmholtz-Centre for Environmental Research, Halle (Saale), Germany; 2 Institute of Biology/Geobotany and Botanical Garden, Martin Luther University Halle-Wittenberg, Halle (Saale), Germany; 3 German Centre for Integrative Biodiversity Research (iDiv) Halle-Jena-Leipzig, Leipzig, Germany; 4 BIOGECO, INRA, Univ. Bordeaux, 33610 Cestas, France; University of Hyogo, JAPAN

## Abstract

Forests with higher tree diversity are often assumed to be more resistant to insect herbivores but whether this effect depends on climatic conditions is so far poorly understood. In particular, a forest’s resistance to herbivory may depend on mean annual temperature (MAT) as a key driver of plant and insect phenology. We carried out a global meta-analysis on regression coefficients between tree diversity and four aspects of insect herbivory, namely herbivore damage, abundance, incidence rate and species richness. To test for a potential shift of tree diversity effects along a global gradient of MAT we applied mixed-effects models and estimated grand mean effect sizes and the influence of MAT, experimental vs. observational studies and herbivores diet breadth. There was no overall effect of tree diversity on the pooled effect sizes of insect herbivore damage, abundance and incidence rate. However, when analysed separately, we found positive grand mean effect sizes for herbivore abundance and species richness. For herbivore damage and incidence rate we found a significant but opposing shift along a gradient of MAT indicating that with increasing MAT diversity effects on herbivore damage tend towards associational resistance whereas diversity effects on incidence rates tend towards associational susceptibility. Our results contradict previous meta-analyses reporting overall associational resistance to insect herbivores in mixed forests. Instead, we report that tree diversity effects on insect herbivores can follow a biogeographic pattern calling for further in-depth studies in this field.

## Introduction

Insect herbivores can compromise the functioning of forest ecosystems [1]. Insect herbivory is controlled by top-down mechanisms involving natural enemies of herbivores [2], bottom-up mechanisms including tree defences [3] and associational effects provided by tree diversity [4, 5]. So-called associational effects occur if herbivory on individual trees is influenced by the identity and density of neighbouring trees [6] and are assumed to be key regulators of herbivory [6, 7]. An increase in tree diversity is often reported to decrease herbivore pressure (associational resistance–AR; [7–9]), but examples for an opposite relationship can also be found (associational susceptibility–AS; [7, 10]).

Associational resistance, on the one hand, occurs if a higher diversity of tree species reduces the availability of host trees for specialist herbivores because of reduced resource availability and reduced encounter rates of herbivores and hosts (resource dilution effects; [11, 12]). Mixtures of host and non-host tree species can also reduce the success of herbivores to detect suitable resources, both visually [13] and olfactorily [14].

Associational susceptibility, on the other hand, occurs if a population of herbivores builds up on a more preferred host species and then ‘spills over’ to admixed less palatable species [10, 15]. Generalist herbivores might furthermore benefit from a more diversified nutrition [16, 17] as suggested by the dietary mixing hypothesis [18, 19].

When accounting for the whole community of herbivores that are able to attack a particular tree, any change in the proportion of generalist vs. specialist herbivores may then shift the balance between AS and AR. Yet, the proportion of specialized insect herbivores was reported to increases towards lower latitudes [20]. As such, it is likely that the direction of associational effects changes along latitudinal gradients.

Several other key features of plant-herbivore interactions are known to vary with latitude. Numerous studies also found that global gradients exist for plant species richness [21, 22], herbivore species richness [23–25], plant defences [26, 27], herbivore pressure [25, 28, 29], leaf herbivory [30] and trophic interactions [31]. Since temperature is a key driver of herbivore development and abundance [32], consumption rates [33] and host-plant choices [34] it is likely to influence these plant-herbivore interactions [33, 34]. Yet, and surprisingly, it still remains to be tested whether the direction and strength of associational effects also change along a global gradient of mean annual temperature (MAT).

In addition to the overlooked effect of MAT on associational effects, the current understanding of AR and AS suffers from several methodological and conceptual biases, including a lack of considering diversity gradients, and confusion between functional (i.e. consumption or proportion of attacked tissue) and quantitative (i.e. abundance and species richness) responses of herbivores.

Previous meta-analyses on associational effects in tree stands mainly compared single species (i.e. monocultures) with mixed stands, irrespective of species richness and species evenness [8, 9]. Yet, the relative share of tree species within tree stands may critically change the way herbivores perceive stand quality [8, 35]. To our knowledge, no study so far has investigated whether the strength and direction of associational effects depend on the metric of tree diversity applied. We therefore compiled studies that reported on either the Shannon or Simpson diversity of tree stands reflecting the relative contribution of host concentration and relative frequency (see [6] and [36] for a discussion about host concentration vs. frequency). If species proportions were not available we applied a gradient of tree species richness or, if species richness was not reported, conducted a comparison between single species and mixed tree stands.

Beside the simplification to monoculture-mixture comparisons, the meta-analyses mentioned above [8, 9] also simplified the response of herbivores by pooling studies that reported on the actual damage inflicted and studies that reported on the abundance of insect herbivores, assuming that more herbivores always cause more damage. This assumption mainly holds for some herbivores such as bark-beetles, leaf miners or galls, but is more controversial for most of defoliators. For instance, herbivore abundance and the associated damage are not necessarily correlated [37, 38] and these two aspects have been reported to sometimes respond differently to plant diversity [7]. Beside, how the species richness of insect herbivores depends on tree diversity has not been summarized in previous meta-analyses. Thus, we separately analysed the response of insect herbivores to increasing tree diversity into the response of herbivore damage, abundance, incidence rate (i.e. the proportion of attacked plant tissue) and the species richness of herbivores.

In the present study, we went beyond previous meta-analyses by testing whether the direction and strength of associational effects on forest trees can partly be attributed to the MAT at the study location. Moreover, we asked whether the focus on different aspects of herbivory (i.e. the amount of insect herbivore damage, abundance, incidence rate and species richness) as well as on different metrics of tree diversity (i.e. the Shannon/Simpson diversity, the species richness and the comparison of single species vs. mixed tree stands) can lead to diverging associational effects.

Specifically, we hypothesized that

1. insect herbivory (pooled over herbivore damage, abundance and incidence rate) is negatively related to tree species diversity;
2. insect herbivore damage, abundance, incidence rate and species richness differ in their relationship to tree species diversity;
3. the relationships between the four aspects of insect herbivory (i.e. herbivore damage, abundance, incidence rate and species richness) and tree diversity change with mean annual temperature.

## Material and Methods

### 2.1. Search strategy and inclusion criteria

**Fig 1:**
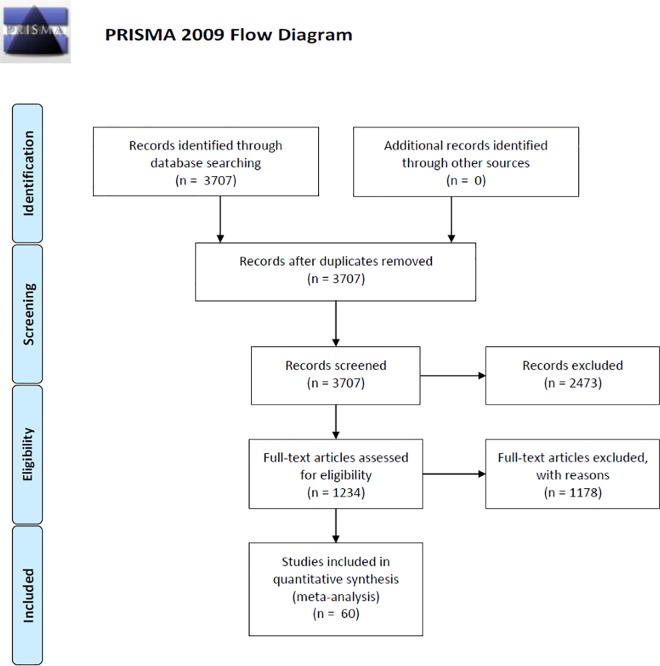
Flow chart of the study search and selection process.

We performed a scoping search with combinations of relevant search terms including the following key words: forest, tree, diversity, richness, herbivores, pest, damage, monoculture, mixture and plantation. The following literature databases were queried in August 2016: Thomson Reuters Web of Science, Google Scholar and Cab Direct. These search queries were partly conducted with the help of the program *Publish or Perish 4* [39]. The PRISMA checklist of items to include when reporting a systematic review is shown in S1 PRISMA checklist [40] and the search protocol used for each database is fully described in S1 Text. After additionally reviewing the literature cited in relevant articles, our search initially yielded 3,707 articles. The search and subsequent selection process is depicted in the flow chart Fig 1.

After the initial screening, we retained 1,234 articles for further in-depth examination. To be included in the present meta-analysis, an article had to report on i) either the Shannon diversity, Simpson diversity, species richness or a monoculture-mixture comparison of tree stands and ii) either the amount of herbivory sustained by trees, the abundance or incidence rate (e.g. the proportion of individuals or parts that were attacked) or species richness of insect herbivores. All causes leading to dismissing articles are fully described in S1 Text.

### 2.2. Effect sizes

Due to the heterogeneity of studies, the compiled measurements of herbivory vastly differed in dimensions and order of magnitude. In the case of herbivore damage, studies reported the percentage of leaf area removed or percentage of crown volume damaged. In the case of herbivore abundance studies reported on the number or density of individuals, galls and egg clusters. Incidences rate were determined as percentage of trees, leaves, or branches attacked or with herbivores present. Species richness was measured as the number of insect herbivore species captured on trees or in traps.

Our main objective was to test the correlation between an increase in tree diversity and the four aspects of tree herbivory (i.e. damage, abundance, incidence rate and species richness). Since Pearson’s correlation coefficient *r* becomes skewed as it approaches ± 1 we transformed *r* to Fisher’s *z*-scores which range from −∞ to +∞, have the same sign as *r* values and are commonly applied in meta-analyses [41–43]. Uncertainty for each effect size was estimated by calculating the corresponding variance estimate (*v* = 1/(*n*-3), where *n* is the sample size [41].

When an article reported multiple measurements (e.g. for different taxa or different aspects of herbivory) we retained them as separate study cases within the same forest and accounted for this non-independence in the statistical analyses (see below).

When studies did not report any *r* value but provided tables or figures with information on tree diversity and any aspect of herbivory we extracted the raw data using the software *ImageJ* [44] and re-calculated the corresponding *r* values. This could only be achieved when there were at least four records for both tree diversity and insect response.

We extracted the mean annual temperature (MAT) for the approximate study locations from the *WorldClim*-global climate data [45]. In order to test for potentially confounding factors, we furthermore distinguished between experimental (i.e. plantations with purposefully manipulated tree diversity) and observational studies (in either semi-natural forests or forests that had not been planted to test for any diversity effects) and classified the diet breadth of herbivores as being either specialists that feed on a single species or genus of tree species or generalists which can utilize a wide range of tree species.

### 2.3. Statistical Analyses

We first tested the grand mean effect size corresponding to the overall effect of increasing tree diversity on the pooled effect sizes of herbivore damage, abundance and incidence rate. Using a random-effect meta-analysis model [43] with restricted maximum likelihood we calculated the model intercept, (i.e. averaged Fisher’s *z*-scores) and the corresponding bootstrap confidence interval (CI). Effect sizes were weighted based on the inverse of their variance estimate. Since multiple effect sizes from the same forest location cannot be considered as fully independent we incorporated a hierarchical random-effect structure with the single effect sizes nested within forest locations. The grand mean effect size was considered statistically significant if the 95% CIs did not include zero. We also estimated the amount of residual heterogeneity (τ^2^; [43]) and tested whether effect sizes displayed significant between-study heterogeneity by applying the weighted Cochran's Q-test.

To test whether effect sizes depended on the aspect of herbivory we pooled all effect sizes on herbivore damage, abundance, incidence rate and species richness of herbivores, included this aspect of herbivory as a moderator in the previous random-effect model and calculated the test statistic for the omnibus test of model coefficients (Q_M_). We then split the dataset based on the aspect of herbivory, applied the intercept only-model from above and calculated for each aspect separately the grand mean effect size, τ^2^ and Q-test statistic.

For each aspect of herbivory, we then tested the correlation between effect sizes and MAT, the type of study (experimental vs. observational) and the diet breadth of insect herbivores.

We thereby included these variables as moderators in the previous random-effect models and tested the significance of the obtained parameter estimates against a normal distribution. To exclude statistically insignificant moderators and to yield robust, most parsimonious models, we performed a backward selection based on error probabilities (*α* = 0.05) calculated with maximum likelihood. The parameter estimates of the resulting four minimal adequate models were then calculated with restricted maximum likelihood. For each model we separately calculated τ^2^, Q_M_ and the test statistic for the amount of residual (i.e. unexplained) heterogeneity (Q_E_, weighted Cochran’s Q-test). The amount of variation in effect sizes that predictors accounted for was finally calculated as Q_M_ / (Q_M_ + Q_E_).

To check for publication bias resulting from the omission of extreme or unlikely results, we visually inspected funnel plots and tested for their asymmetry by applying Egger's regression test [42, 46]. We furthermore tested whether the year of publication correlated with effect sizes or MAT at the study location by calculating Pearson correlation coefficients and the corresponding test statistics. We finally checked whether effect sizes correlated with the reported metric of tree diversity (i.e. Shannon/Simpson diversity, species richness and single vs. mixed species stands) by including this moderator in the previous random-effect model and calculating Q_M_.

All statistical analyses were conducted in *R* [47] using the packages *metafor* for meta-analyses [48], *vegan* for calculating the Shannon diversity [49], *raster* for extracting the *WorldClim* data [50] and *ggplot2* for graphical representations [51].

## Results

The final dataset consisted of 60 studies with 173 study cases that reported on the correlation between tree diversity and insect herbivore damage (53 study cases), abundance (52 study cases), incidence rate (40 study cases) and species richness (28 study cases), respectively. Regarding the metric of tree diversity, 94 and 7 study cases reported the Shannon or Simpson diversity of tree species, respectively, whereas 44 study cases reported the richness of tree species. In the remaining 28 study cases, we could apply a comparison between tree monocultures and mixtures of tree species, without any further quantification of tree species diversity.

In 92 study cases, herbivory was measured on a focal tree species whereas in the remaining 81 cases herbivory and especially herbivore abundance and species richness were measured at the plot level, either by summarizing herbivory over all tree species or by reporting capture rates in insect traps that could not be related to certain tree species. All study cases and study characteristics are documented in S1 File.

**Fig 2:**
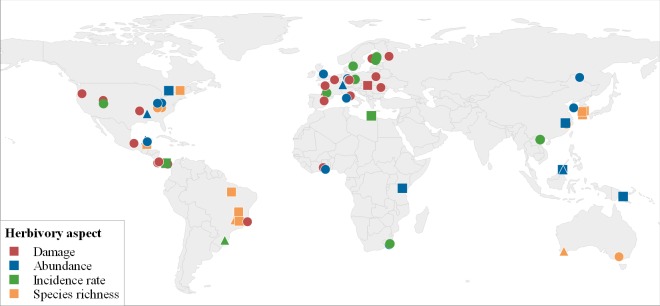
Locations of the studies included in this meta-analysis reporting on the relationship between the four aspects of insect herbivory and tree Shannon/Simpson diversity (circles), tree species richness (squares) or the comparison between single vs. mixed stands (triangles). The colouring indicates the different aspects of insect herbivory. Made with Natural Earth. Free vector and raster map data (naturalearthdata.com).

The included study cases covered a latitudinal range of -36.7° to 62.8° and spanned a gradient of -3.3°C to 26.9°C mean annual temperature (MAT). Study sites were most frequent in Europe and North to Middle America and sparse in Asia, Africa and Australia (Fig 2).

Pooled over all study cases that reported herbivore damage, abundance and incidence rate, the grand mean effect size was not significant and the corresponding funnel plot was symmetric (Table 1, S2 Text, Egger’s test: p = 0.58). There was a significant amount of residual heterogeneity indicating that heterogeneity in true effect-sizes could be accounted for by moderators.

**Table 1:** Intercept-only and most parsimonious mixed-effects meta-regression models.

Model	Model statistics			Parameters	Mean	Se	z-value	p-value
**Grand mean all**								
(= damage, abundance and	τ^2^ = 0.11			Intercept	0.01	0.03	0.31	0.76
incidence rate pooled)	Q = 395.35	df = 144	p < 0.001					
**Damage**								
*Grand mean*	τ^2^ = 0.07	AICc = 67.03		Intercept	0.06	0.06	1.12	0.26
	Q = 92.24	df = 52	p < 0.001					
*Most parsimonious model*	τ^2^ = 0.07	AICc = 65.63		Intercept	0.14	0.1	1.33	0.18
				(observational at zero MAT)				
	Q_E_ = 77.99	df = 50	p = 0.01	**MAT**	**- 0.02**	**0.01**	**- 2.3**	**0.02**
	Q_M_ = 8.22	df = 2	p = 0.02	**Experimental vs.**	**0.23**	**0.1**	**2.24**	**0.03**
				**observational**				
**Abundance**								
*Grand mean*	τ^2^ = 0.19	AICc = 93		**Intercept**	**0.08**	**0.04**	**2.13**	**0.03**
	Q = 220.33	df = 51	p < 0.001					
*Most parsimonious model*	τ^2^ = 0.15	AICc = 92.17		**Intercept**	**0.15**	**0.08**	**1.94**	**0.05**
	Q_E_ = 210.51	df = 50	p < 0.001	**(generalist)**				
	Q_M_ = 3.64	df = 1	p = 0.06	**Specialists vs**	**- 0.26**	**0.14**	**- 1.91**	**0.06**
				**generalists**				
**Incidence rate**								
*Grand mean*	τ^2^ = 0.06	AICc = 56.44		Intercept	- 0.08	0.07	- 1.08	0.28
	Q = 73.18	df = 39	p < 0.001					
*Most parsimonious model*	τ^2^ = 0.04	AICc = 43.54		**Intercept**	**- 0.42**	**0.07**	**- 5.67**	**< 0.001**
	Q_E_ = 57.75	df = 38	p = 0.02	**MAT**	**0.03**	**0.01**	**4.9**	**< 0.001**
	Q_M_ = 23.97	df = 1	p < 0.001					
**Species richness**								
*Grand mean*	τ^2^ = 0.34	AICc = 57		**Intercept**	**0.36**	**0.15**	**2.35**	**0.02**
(= most parsimonious model)	Q = 99.23	df = 27	p < 0.001					

Mixed-effects models tested the effect of mean annual temperature (MAT), study design (tree plantations or semi-natural forests) and herbivore specialization on the transformed correlation coefficient (Fisher’s *z*-scores) between the diversity of tree species and the four different aspects of herbivory. In each model the intercept denotes the reference level of coefficient estimates, τ^2^ denotes the variance between study cases, Q/Q_E_ relate to Cochran's Q-test for residual heterogeneity and Q_M_ denotes to the omnibus test of model coefficients. Significant parameter estimates are in bold.

**Fig 3:**
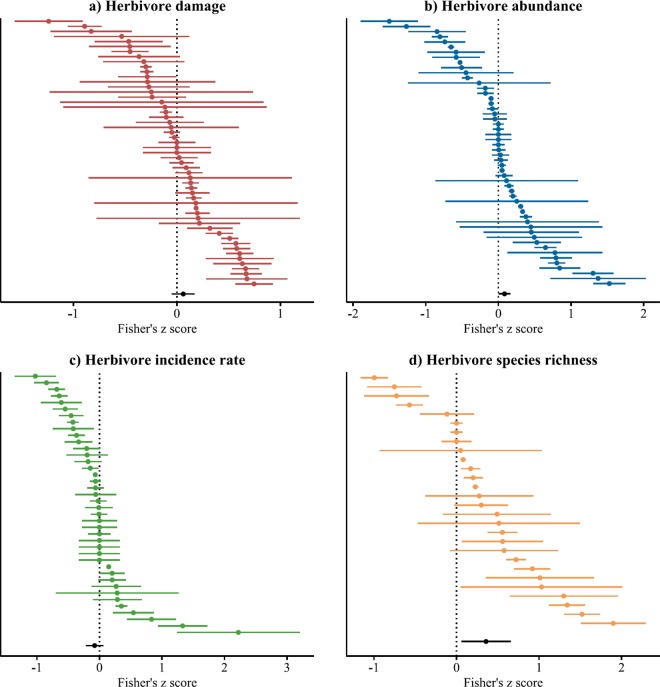
Forest plots for the transformed correlation coefficients (Fisher’s *z*-scores) between tree diversity and a) the damage sustained by, b) the abundance of, c) the incidence rate of, d) the species richness of insect herbivores. Each point represents the Fisher’s *z*-score and the approximated confidence interval (= mean ± standard error × 1.96) for an individual study case. Negative values indicate associational resistance while positive values indicate associational susceptibility. Grand mean effect sizes, together with their 95% bootstrap confidence intervals, are shown in black at the bottom of each forest plot.

Across the whole dataset (including effect sizes on herbivore species richness) the aspect of herbivory was a significant moderator of effect sizes as indicated by the significant omnibus test of model coefficients (Q_M_ = 10, df = 3, p = 0.02). When analysed separately, study cases on herbivore abundance and species richness yielded significantly positive grand mean effect sizes whereas herbivore damage and incidence rate showed no significant relationship with increasing tree diversity (Table 1, Fig 3). Funnels plots for these separate models were symmetric following Egger’s regression test (damage: p = 0.14, abundance: p = 0.7, species richness: p = 0.25) except for herbivore incidence rate that displayed a positive correlation between effect sizes and the corresponding variance estimates (estimated effect sizes: 2.1 ± 0.9 standard error, p = 0.02).

**Fig 4:**
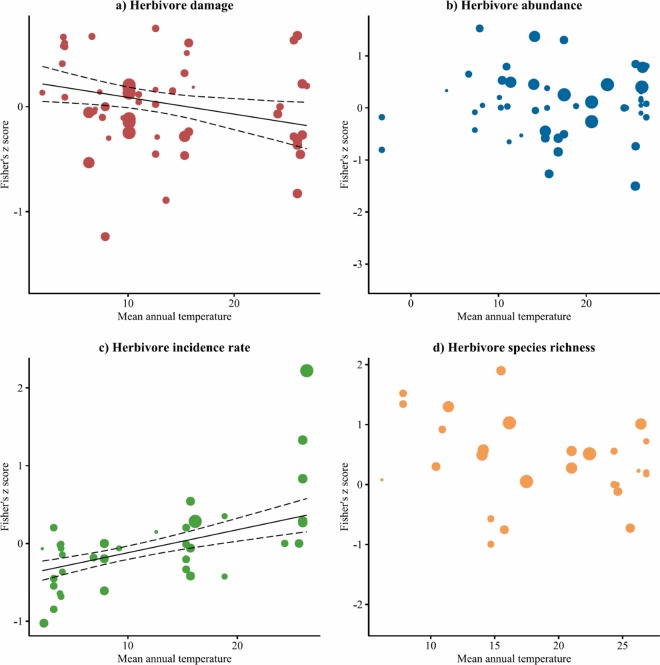
Relationships between mean annual temperature (MAT) and the transformed correlation coefficients (Fisher’s *z*-scores) between the diversity of tree species and a) the damage sustained by, b) the abundance of, c) the incidence rate of, d) the species richness of insect herbivores. Each point represents an individual study case for which negative values indicate associational resistance while positive values indicate associational susceptibility. The size of each point indicates its weight for estimating the regression slope (solid line) and the corresponding approximated credible interval (dotted lines) in a mixed-effects meta-regression model. Coefficient estimates for MAT are reported in Table 1.

Effect sizes for herbivore damage showed a decrease with MAT, indicating a tendency toward associational resistance in warm regions, and associational susceptibility in cold regions (Fig 4), and were negative in experimental (estimated effect size: -0.02 ± 0.08, Table 1) and positive in observational studies (0.16 ± 0.11, Table 1). The abundance of generalist herbivores increased with tree diversity (estimated effect size: 0.15 ± 0.08, Table 1) while it decreases for specialist herbivores (-0.12 ± 0.1, Table 1). The included predictors explained 9.5 and 1.6 percent of variation for herbivore damage and abundance, respectively. As for herbivore incidence rate, effect sizes displayed a significantly positive relationship with MAT which accounted for 29.3 percent of variation. Effect sizes for herbivore species richness were not related to any of the proposed predictors (Table 1, Fig 4).

Addressing publication bias, most of funnel plots were symmetric, indicating that studies reporting positive and negative correlations, with low and high sample sizes were equally likely to be published (S2 Text). The omnibus test of model coefficients indicated that effect sizes did not depend on the metric of tree diversity applied (Q_M_ = 5.66, df = 3, p = 0.129; S2 Text). We found a negative correlation between the year of publication and MAT (r = -0.18, p = 0.01), showing that studies in cooler climates were carried out later, but not between the year of publication and the actual effect sizes (r = -0.01, p = 0.87), indicating that there was no temporal shift in our understanding of associational effects.

## Discussion

Our meta-analysis does not support previous claims of pervasive, globally consistent associational resistance (AR) to insect herbivores in mixed forests [8, 9]. Importantly, we show that several sources of variation in the strength and direction of associational effects have been overlooked, in particular mean annual temperature (MAT). We found no negative relationship between tree diversity and herbivory, regardless if the dataset was split according to the aspect of herbivory or the metric of tree diversity and, thus, have to reject our first hypothesis.

Instead, and in confirmation of our second hypothesis, we demonstrated that grand mean effect sizes differed between the four aspects of herbivory. Notably, herbivore species richness, an aspect not analysed in previous syntheses, increased with tree diversity. Importantly, we detected a significant relationship between MAT and the response of herbivore damage and incidence rate to increasing tree diversity. This finding partly confirms our third hypothesis and strongly improves our understanding of heterogeneity among studies.

We found that both aspects of herbivory displayed quite opposite tendencies. With increasing MAT, diversity effects on herbivore damage shifted from associational susceptibility (AS) in cold regions towards AR in warmer regions, whereas the opposite pattern was encountered for herbivore incidence rate, with a tendency towards AS in warmer regions.

As our study was based on correlation coefficients which become significant if any increase in tree diversity is accompanied by a steady increase or decrease in herbivory, we cannot make any statement on the change in the absolute amount of herbivory. However, we demonstrated that future syntheses on the strength of associational effects in forest systems should differentiate between the aspects of herbivory and consider the environmental context of the study site. This is critical because different aspects of tree diversity may influence damage (i.e. actual consumption) and incidence rate. For instance, incidence rate could reflect herbivore foraging behaviour, while damage additionally could include tree diversity effects on food quality and herbivore survival.

In search for factors that underlie the documented biogeographic gradient, we ask for more research on how associational effects depend on and follow global gradients in i) regional species diversity of herbivores and host plants, ii) herbivore density and pressure, especially during times of outbreaks [25, 28–30, 52–56], iii) herbivore specialization [4, 5, 8, 9, 20, 57], iv) tree defences [25, 27, 29, 31, 52, 58] and v) abiotic factors affecting tree or herbivore development (e.g. precipitation, climate stability, CO_2_-concentration, UVB-radiation).

The literature on global gradients in herbivore pressure is controversial, providing examples of either increasing [25, 29, 52–54], unaffected [25, 29, 53, 55] or decreasing [28, 56] pressure towards warmer climates. Regarding the level of background herbivory, e.g. the regular loss of woody plant foliage, a recent global analysis even pointed out that background herbivore pressure could show a nonlinear global pattern that is peaking in temperate regions [30].

The highest levels of herbivore pressure can be studied during insect outbreaks when herbivores face strong intraspecific competition and resource depletion. If, during outbreaks, herbivorous individuals are forced to forage less selectively, this might shift associational effects from AR to AS, regardless of the composition of tree species mixtures. Unfortunately, our dataset included only 5 study cases (3 original studies) that measured herbivory during outbreaks (S1 File) and we are not aware of any study that investigated global gradients in the frequency and intensity of insect herbivore outbreaks in forest ecosystems.

Global patterns in the relative abundance of generalist vs. specialist insect herbivores, however, had already been addressed. Here, Novotny et al. [59] and Schuldt et al. [4] proposed that tropical and subtropical forest are dominated by generalist herbivores, which, according to previous meta-analyses [8, 9], are not affected or even benefit from the diversification of tree stands. A higher proportion of generalist species could explain the shift in herbivore incidence rates from AR to AS with increasing MAT. However, more comprehensive and global analyses concluded that the proportion of generalist species actually decreases towards tropical regions [20, 57].

Beside the difficulty to relate the documented shifts in associational effects with MAT to a single, underlying factor, any global synthesis of plant-plant-herbivore interactions might furthermore be confronted with gradients that are non-linear, non-additive or interrelated (i.e. show interactions such as latitudinal changes in plant defences being counterbalanced by higher herbivore pressure [29, 52]). In addition to the abiotic and biotic factors already mentioned, many decisions on the design of a study, such as the spatial scale of the investigated plant neighbourhood [60], the age of the forest stand [61] and the sampling date [62] are likely to determine the sampling success of insect herbivore communities and thus impact the conclusions on the direction and strength of associational effects.

Given the multitude of potentially confounding factors, it is hardly surprising that our study documented a high amount of unexplained heterogeneity, and thus, highlights the limits of our meta-analytical approach. Here, research co-operations, such as the globally distributed network of tree diversity experiments (www.treedivnet.ugent.be), can offer great future opportunities to experimentally study associational effects along replicated global abiotic and biotic gradients [63].

## Conclusion

Our results indicate that studies of associational effects on herbivory might need to consider the biogeographical context in which plant-plant-herbivore interactions occur. Yet, along such gradients, joint impacts of insect herbivore diversity, pressure, specialization and abiotic factors on a global gradient of associational effects are difficult to disentangle.

We recommend that information on the damage, incidence, abundance and diversity, together with the identity of herbivores, be systematically recorded in observational and experimental tree diversity studies to provide a sounder understanding of mechanisms involved in AR and AS.

The inability to replicate major findings of previous meta-analyses, namely the significance of AR of mixed tree stands to insect herbivory [8, 9], could even indicate that associational effects are nonlinear, thus adding another layer of complexity.

A better understanding of the mechanisms at play will require addressing the identity, functional characteristics, density and diversity of both, insect herbivores and focal tree species, in a systematic way along large geographical gradients.

## Supporting Information

S1 PRISMA Checklist (DOC)

S1 File Coding table for this analysis. Including a list of publications and researchers that provided the data for this meta-analysis.
(XLSX)

S1 Text Search protocol. (DOCX)

S2 Text Funnel plot diagrams. (DOCX)
