# Supplementary material for: The Impact of Tree Diversity on Different Aspects of Insect Herbivory along a Global Temperature Gradient - A Meta-Analysis
Source: PLoS One. 2016 Nov 11;11(11):e0165815. doi: 10.1371/journal.pone.0165815 (PMC5105991; doi:10.1371/journal.pone.0165815)
Supplement: S1 Text — (DOCX) [file pone.0165815.s003.docx]

**1. Search Strategy**

On August 1st, 2016 we searched the following databases for relevant literature:

*Thomas Reuters Web of Knowledge*

We retrieved all literature listed for the following search term:

- "forest" AND "tree" AND (herbivor* OR pest*) AND (diversit* OR richness OR monocultur* OR mixtur* OR polycultur* OR plantatio*)

*Google Scholar*

The search was performed with the program *Publish or Perish 4*.

We retrieved the first 1000 entries from the following combinations of search terms:

- All of the words: *Forest Tree Diversity Herbivory*
- Any of the words: *Richness Damage Rate Monocultures Mixtures Polycultures*
- All of the words: *Forest Tree Monocultures Mixtures Herbivores*
- Any of the words: *Diversity Damage Rate Polycultures*
- All of the words: *Forest Herbivore Resistance Diversity Tree Richness*
- Any of the words: *Monoculture Plantations Mixture Insects*

*Cabdirect*

We retrieved all entries from the following combinations of search terms:

- *forest AND tree AND herbivor* AND diversity OR richness OR monocultur* OR mixtur* OR polycultur* OR plantatio* OR damage*

**2. Study Inclusion Criteria**

*Relevant subject(s)*

We included all studies conducted in forests or forest plantations that reported either the Shannon/Simpson diversity, species richness or a comparison of monocultures and mixtures of tree species per plot together with the corresponding information on one of the following aspects of insect herbivory: damage inflicted on trees, abundance/density on trees or in traps, incidence rate (proportion of trees or tree parts attacked or occupied by herbivores) or the species richness of herbivores collected on trees or in traps.

##### *Types of Intervention*

We considered studies that i) manipulated tree species diversity in experimental plantings, ii) compared monocultures with stands of higher tree species richness or iii) compared proximal forest stands of varying tree species diversity. Studies that compared tree stands with varying densities of trees planted were not included into the analyses for not confounding any diversity with density effects.

##### *Types of Comparator*

i) Damage on trees inflicted by insect herbivores

ii) Abundance/density of herbivorous insects species

iii) Incidence rate of herbivorous insect species

 iii) Species richness of herbivorous insect species

**3. Assessment of Study Relevance**

Studies were not included into the analysis for the following reasons:

- No established forest
  - Studies were not conducted in tree stands but rather on single individuals, botanical gardens, mixed landscapes, etc.
- No comparison of tree diversity levels
  - Compared sites had the same diversity of tree species
- No herbivory reported
  - Study did report neither of the four focal aspects of insect herbivory
- Not a primary study
  - Reviews, opinion papers, conceptual papers,simulation models, etc… that did not report experimental or observational data
- Paper not found/no access
- Manipulation of herbivore pressure
  - Studies manipulated herbivory and assessed consequences on tree diversity
- Focus on soil interactions
- Unclear herbivory measurement
  - It was not possible to assign herbivory to either damage, abundance, incidence rate or species richness
- More suitable study on the same site
  - A more recent or a more comprehensive study was published from the same sites
- Forest plots are not comparable
  - Forest plots differ in land-use, succession status, environmental conditions, tree density or fragment size so that comparisons of associational effects are likely flawed
- Seed survival reported
