# Supplementary material for: The Impact of Tree Diversity on Different Aspects of Insect Herbivory along a Global Temperature Gradient - A Meta-Analysis
Source: PLoS One. 2016 Nov 11;11(11):e0165815. doi: 10.1371/journal.pone.0165815 (PMC5105991; doi:10.1371/journal.pone.0165815)
Supplement: S2 Text — (DOCX) [file pone.0165815.s004.docx]

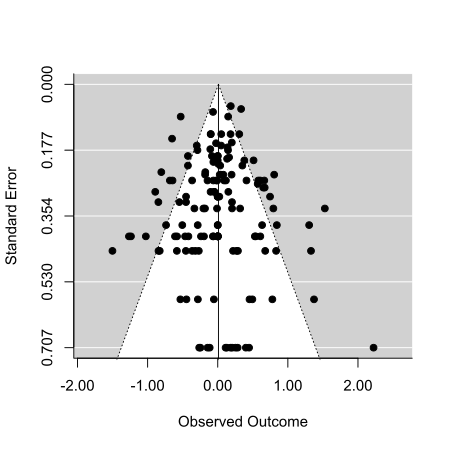


**Figure S4.1**: Funnel plot for the combined response different measures of insect herbivory (damage, abundance and incidence rate) to an increase in tree diversity. Observed outcome refers to transformed correlation coefficients (Fisher’s *z*-scores). The vertical line represents the grand mean effect size which is not significantly different from zero.


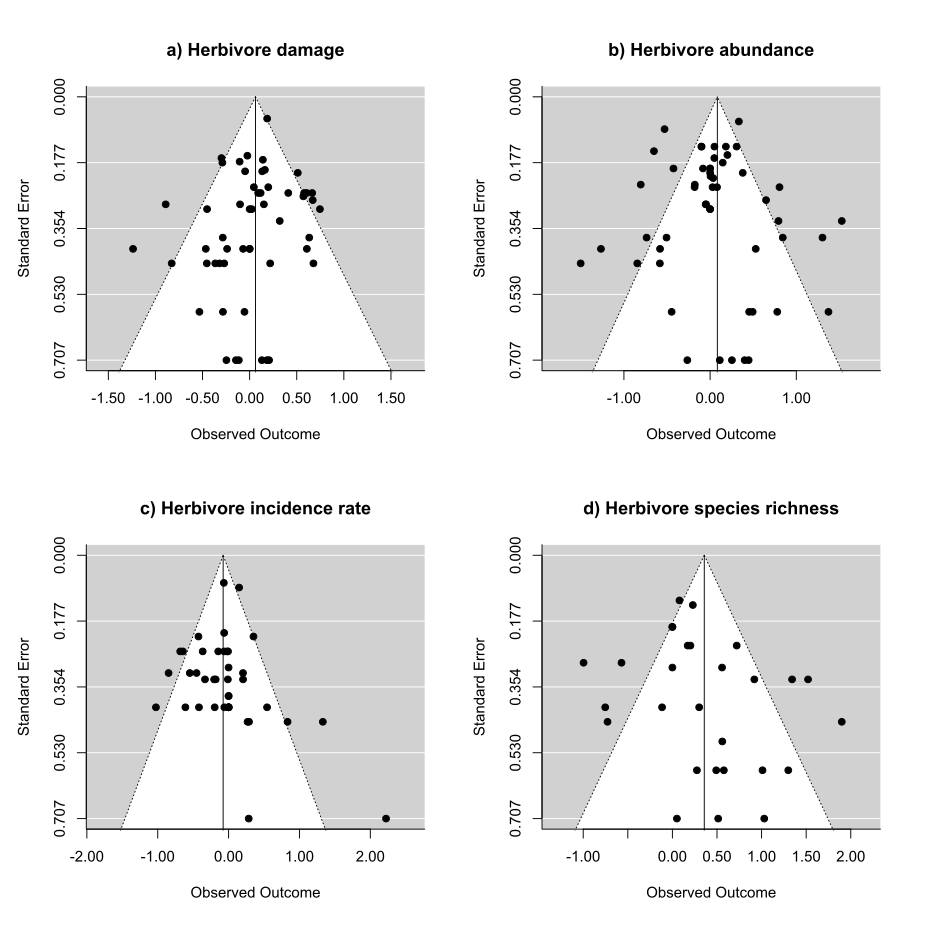


**Figure S4.2**: Funnel plots for the response of different measures of insect herbivory to an increase in tree diversity. Observed outcome refers to transformed correlation coefficients (Fisher’s *z*-scores). The vertical line represents the grand mean effect size which is statistically significant from zero for the response of herbivore abundance and species richness.


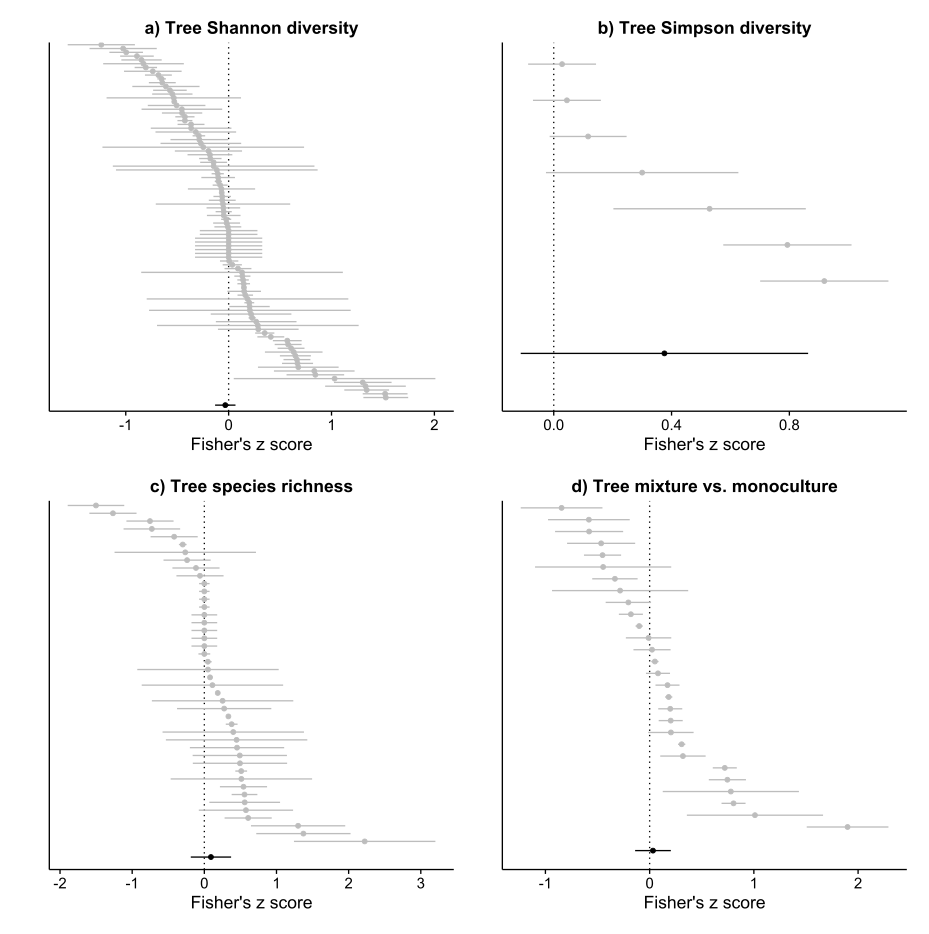


**Figure S4.3**: Forest plot for the transformed correlation coefficients (Fisher’s *z*-scores) between insect herbivory (damage, abundance and incidence rate combined) and a) the Shannon diversity, b) the Simpson diversity, c) the species richness and d) a single and mixed stands of forest trees. Each point represents the mean Fisher’s *z*-score and the approximated confidence interval (= mean + standard error * 1.96) for a single study case. Negative values indicate associational resistance while positive values indicate associational susceptibility. Grand mean effect sizes together with their 95% bootstrap confidence intervals are shown at the bottom of each forest plot.
